# Poxvirus Targeting of E3 Ligase β-TrCP by Molecular Mimicry: A Mechanism to Inhibit NF-κB Activation and Promote Immune Evasion and Virulence

**DOI:** 10.1371/journal.ppat.1003183

**Published:** 2013-02-28

**Authors:** Daniel S. Mansur, Carlos Maluquer de Motes, Leonie Unterholzner, Rebecca P. Sumner, Brian J. Ferguson, Hongwei Ren, Pavla Strnadova, Andrew G. Bowie, Geoffrey L. Smith

**Affiliations:** 1 Department of Virology, Faculty of Medicine, Imperial College London, St Mary's Campus, London, United Kingdom; 2 Department of Pathology, University of Cambridge, Cambridge, United Kingdom; 3 School of Biochemistry and Immunology, Trinity Biomedical Sciences Institute, Trinity College Dublin, Dublin, Ireland; University of Alberta, Canada

## Abstract

The transcription factor NF-κB is essential for immune responses against pathogens and its activation requires the phosphorylation, ubiquitination and proteasomal degradation of IκBα. Here we describe an inhibitor of NF-κB from vaccinia virus that has a closely related counterpart in variola virus, the cause of smallpox, and mechanistic similarity with the HIV protein Vpu. Protein A49 blocks NF-κB activation by molecular mimicry and contains a motif conserved in IκBα which, in IκBα, is phosphorylated by IKKβ causing ubiquitination and degradation. Like IκBα, A49 binds the E3 ligase β-TrCP, thereby preventing ubiquitination and degradation of IκBα. Consequently, A49 stabilised phosphorylated IκBα (p-IκBα) and its interaction with p65, so preventing p65 nuclear translocation. Serine-to-alanine mutagenesis within the IκBα-like motif of A49 abolished β-TrCP binding, stabilisation of p-IκBα and inhibition of NF-κB activation. Remarkably, despite encoding nine other inhibitors of NF-κB, a VACV lacking A49 showed reduced virulence in vivo.

## Author Summary

The host response to infection provides a powerful means of restricting the replication and spread of viruses. Consequently, viruses have evolved mechanisms to reduce activation of host response to infection and this paper provides an example of this. Nuclear factor kappa B (NF-κB) is an important transcription factor that activates the host response to infection and is normally retained in an inactive form in the cytoplasm bound to an inhibitor called IκBα. However, upon stimulation by infection, IκBα is degraded and NF-κB moves to the nucleus to activate expression of genes mediating the host response. Here we describe how protein A49 from vaccinia virus, the vaccine used to eradicate smallpox, mimics IκBα to hijack the cellular degradation machinery and so stabilise IκBα and retain NF-κB in the cytoplasm. The importance of A49 is demonstrated by the fact that a virus lacking A49 was less virulent than control viruses, despite the expression of several other NF-κB inhibitors by vaccinia virus. Interestingly, HIV protein Vpu functions in a similar way to A49 and, given that A49 is highly conserved in variola virus, this work reveals a common strategy for suppression of host innate immunity by the viruses that cause smallpox and AIDS.

## Introduction

Mammals respond to infection by activation of innate and adaptive immunity. In the past two decades, the discovery of pattern recognition receptors (PRRs) such as Toll-like receptors (TLR), intracellular nucleic acid sensors and inflammasomes has established the link between sensing pathogens and responding to them [1]. Following sensing of pathogen associated molecular patterns (PAMPs), signalling cascades lead to the activation of transcription factors that induce the expression of interferons (IFN), cytokines, chemokines and other pro-inflammatory molecules. Nuclear factor kappa B (NF-κB) is a transcription factor that plays a central role in switching on the immune system and proteins induced by NF-κB are responsible for the amplification of the innate response and for the recruitment of cells of the immune system, so linking innate and adaptive immunity [2], [3].

The signalling cascade leading to transcriptional activation of pro-inflammatory genes by NF-κB is well studied [4]. It can be initiated by TLR ligands, interleukin (IL)-1 or tumour necrosis factor (TNF)α and leads to the phosphorylation of the inhibitor of κB (IκB) by the IκB kinase (IKK) complex, the ubiquitination and degradation of phosphorylated IκB (p-IκB), and the translocation of the NF-κB heterodimer p65/p50 into the nucleus [5]. These steps are central in the NF-κB canonical signalling pathway and represent targets for pathogen evasion [6]. In particular, the ubiquitination and degradation of IκBα requires the recognition of its phosphorylated form [7] by the E3 ligase β-transducing repeat containing protein (β-TrCP) [8]. β-TrCP belongs to the Skp1, Cullin1, F-box protein (SCF) family and induces IκBα ubiquitination [9], [10]. β-TrCP was identified originally as the ubiquitin ligase targeted by human immunodeficiency virus (HIV)-1 viral protein U (Vpu) to cause CD4 degradation [11] and exists in 2 forms with very similar properties and specificity [12].

Poxviruses are large DNA viruses that replicate in the cytoplasm [13]. The prototypal poxvirus, vaccinia virus (VACV), was the live vaccine used to eradicate smallpox [14]. Poxviruses express many immunomodulatory proteins, encoded in the terminal regions of the genome [15], that can synthesise immunosuppressive steroids [16], [17] or block the production or action of cytokines, chemokines, IFNs and complement, for reviews see [18]–[20]. For instance, several VACV proteins inhibit activation of NF-κB: protein A52 binds TNF receptor associated factor 6 (TRAF6) and IL-1 receptor associated kinase 2 (IRAK2) and inhibits NF-κB activation downstream of TLRs and the IL-1 receptor [21], [22]. Protein A46 binds to different TLR adaptor molecules [21], [23]. Protein B14 binds to IKKβ and thereby reduces phosphorylation of IκBα [24]–[27]. Protein N1 inhibits NF-κB activation downstream of TRAF6 [28], [29] and protein M2 reduced p65 nuclear translocation [30]. Proteins K7 and K1 inhibit NF-κB activation by either inhibiting TLR-induced signalling (K7) [31] or by blocking IκBα degradation (K1) [32]. Protein E3 inhibits NF-κB activity [33] and antagonizes the RNA polymerase III-dsDNA-sensing pathway [34]. Lastly, protein C4 inhibits NF-κB at or downstream of the IKK complex [35]. However, genetic evidence predicts additional VACV inhibitor(s) of NF-κB activation that stabilise phosphorylated IκBα (p-IκBα) [36].

Here we show that VACV protein A49 is an inhibitor of NF-κB activation that contributes to virus virulence. Like HIV Vpu, A49 exploits molecular mimicry of p-IκBα to bind to the E3 ubiquitin ligase β-TrCP. Consequently, p-IκBα is not ubiquitinated or degraded and so remains in complex with the NF-κB (p65/p50) complex in the cytoplasm. A highly conserved counterpart of VACV A49 is encoded by variola virus, suggesting that the pathogens that cause smallpox and AIDS have evolved a common strategy to suppress innate immunity.

## Results

### A49 is a virulence factor

A screen of genes near the VACV genome termini for proteins that inhibited the induction of the IFNβ promoter led to the discovery of proteins C6 [37] and A49, the subject of this paper.

The *A49R* gene is near the right genome terminus between the genes encoding thymidylate kinase [38] and DNA ligase [39] and is predicted to encode an 18.8-kDa protein, which is conserved in other VACV strains and orthopoxviruses including variola virus [40] (Figure S1A) but not in monkeypox, camelpox and ectromelia viruses where the coding region is disrupted (www.poxvirus.org). However, outside poxviruses no clear counterparts were identified by bioinformatic searches. The *A49R* gene is transcribed both early and late during infection [41]. Consistent with this, an A49 antibody (Methods) detected the A49 protein in VACV strain Western Reserve (WR)-infected cells within 2 h of infection and in the presence of cytosine arabinoside (AraC), an inhibitor of DNA and late protein synthesis (Figure S1B). At later time points A49 expression was reduced by AraC indicating late expression also (Figure S1B), and this is consistent with a TAAAT motif upstream of the A49 open reading frame (ORF) that is a feature of VACV late promoters [42].

To study the role of the A49 protein in virus replication, a VACV WR strain in which the *A49R* ORF was deleted (vΔA49), and a revertant control with the *A49R* ORF reinserted (vA49rev) were constructed. These virus genomes were analysed by PCR and restriction enzyme digestion, and no differences were seen except at the *A49R* locus of vΔA49 (data not shown). These viruses had indistinguishable growth curves (Figure S2A and S2B) and ability to form plaques (Figure S2C) showing A49 is not essential for replication *in vitro*.

**Figure 1:**
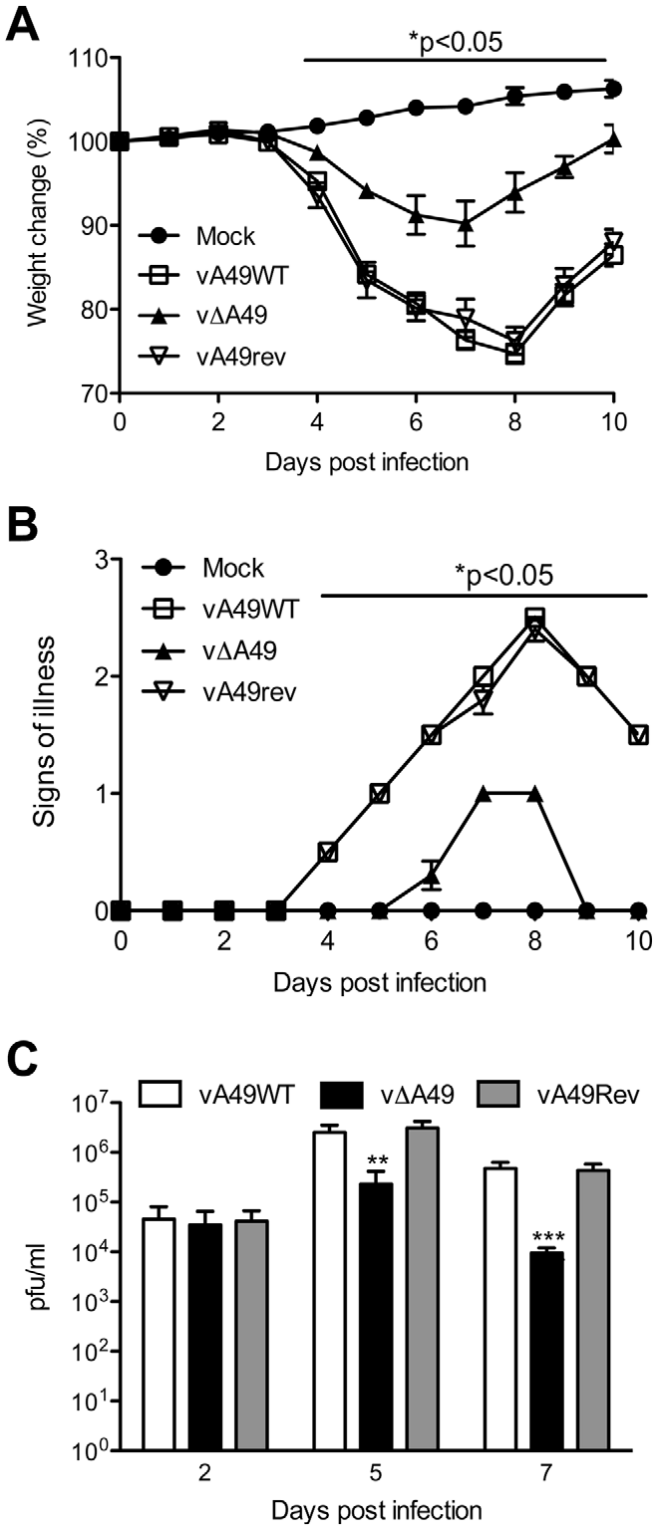
A49 is a virulence factor. Groups of 5 BALB/c mice were infected intranasally with 5×10^3^ PFU of the indicated viruses and their weights (A) and signs of illness (B) were monitored daily. Weights are expressed as the percentage ± SEM of the mean weight of the same group of animals on day 0. Signs of illness (B) are expressed as the mean score ± SEM. (C) At day 2, 5 and 7 pi, lungs from infected animals were extracted and virus titres were assessed by plaque assay. Statistical significance is indicated by horizontal bars after analysis with one-way ANOVA with Friedman and Dunn's multiple comparison test (A), or unpaired t-test comparing WT with vΔA49 (B–C). Data correspond to one representative experiment out of two showing indistinguishable results.

The contribution of A49 to virulence was tested by infecting groups of BALB/c mice intranasally and measuring weight loss and signs of illness [43]. Animals infected with vΔA49 lost less weight and recovered more quickly than controls (Figure 1A) and showed fewer signs of illness on days 4 to 10 post infection (pi) (Figure 1B). Measurement of infectious virus in lungs showed that all viruses replicated to similar titres by day 2 pi, but on days 5 and 7 pi mice infected with vΔA49 had significantly lower titres, showing more rapid clearance of virus (Figure 1C). Collectively, these data indicate that A49 is non-essential for replication, but is a virulence factor. The degree of attenuation seen by deletion of the A49 gene is similar to that deriving from deletion of many other VACV immunomodulators in this model [16], [17], [22], [23], [35], [37], [43]–[50].

### A49 inhibits IFNβ and CCL5 expression

**Figure 2:**
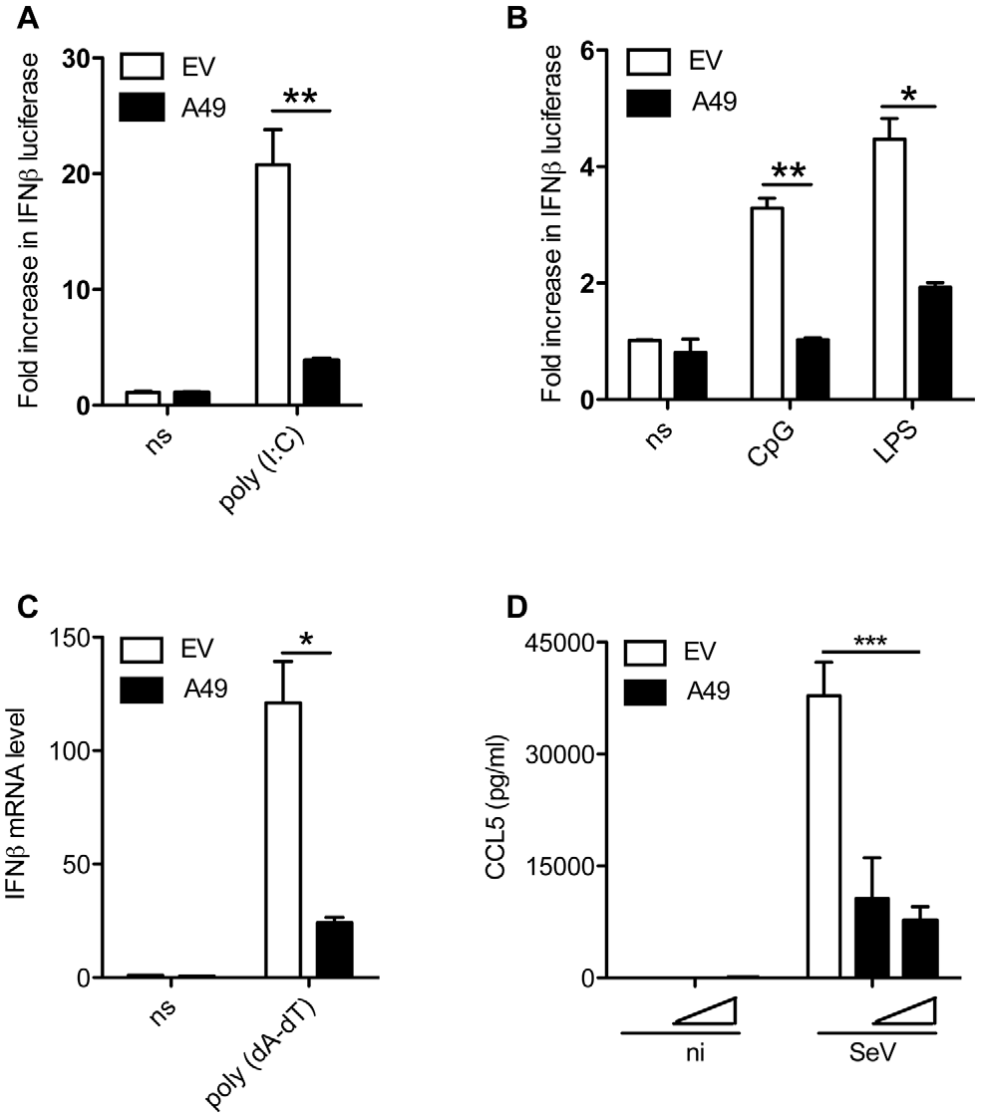
A49 inhibits early innate immune signalling events. (A) HEK293T cells were transfected with an IFNβ-promoter luciferase reporter, a TK-promoter renilla luciferase transfection control, TLR3, and pCI-A49 or empty vector (EV). After 24 h cells were stimulated with 100 µg/ml of poly(I∶C) for 6 h before luciferase activity was measured. (B) RAW 264.7 cells were transfected as in (A) and stimulated with CpG or LPS for 6 h before luciferase activity was measured. (C) HEK293T cells were transfected with pCMV-HA-A49 or EV and stimulated with 500 ng/ml of poly (dA-dT) for 24 h. RNA was extracted and IFNβ mRNA measured by real-time PCR. (D) HEK293T cells were transfected as in (C) with different doses of A49 plasmid and infected 24 h later with Sendai virus (SeV) for further 24 h. CCL5 was measured by ELISA in the supernatant of infected and mock-infected cells. NI stands for non-infected, NS for non-stimulated. In all assays, data are presented as mean ± SD and show one representative experiment of at least three, each performed in triplicate. *p<0.05, **p<0.01 or ***p<0.001 comparing A49 transfected cells with EV.

Next, the mechanism by which A49 inhibited activation of the IFNβ promoter was studied. The *A49R* ORF was amplified from VACV WR genomic DNA and cloned into a mammalian expression vector with a C-terminal Flag or an N-terminal HA tag and tested for inhibition of the IFNβ pathway. HEK293 cells were co-transfected with an IFNβ promoter-firefly luciferase reporter and an A49 expression vector or the empty vector (EV), TLR3 (to allow IFNβ induction by poly(I∶C)) and a renilla luciferase transfection control. Cells were stimulated 24 h later with poly(I∶C), poly(dA∶dT) or infected with Sendai virus (SeV) (Figure 2). A49 blocked activation of the IFNβ promoter by poly(I∶C) (Figure 2A). The same effect was seen in RAW 264.7 cells stimulated with LPS and CpG, agonists of TLR4 and TLR9, respectively (Figure 2B). A49 also diminished transcription of IFNβ mRNA in poly(dA-dT)-stimulated HEK293T cells, as shown by quantitative PCR (Figure 2C), and inhibited production of the NF-κB responsive chemokine CCL5 in SeV-infected HEK293T cells (Figure 2D).

### A49 inhibits NF-κB activation

**Figure 3:**
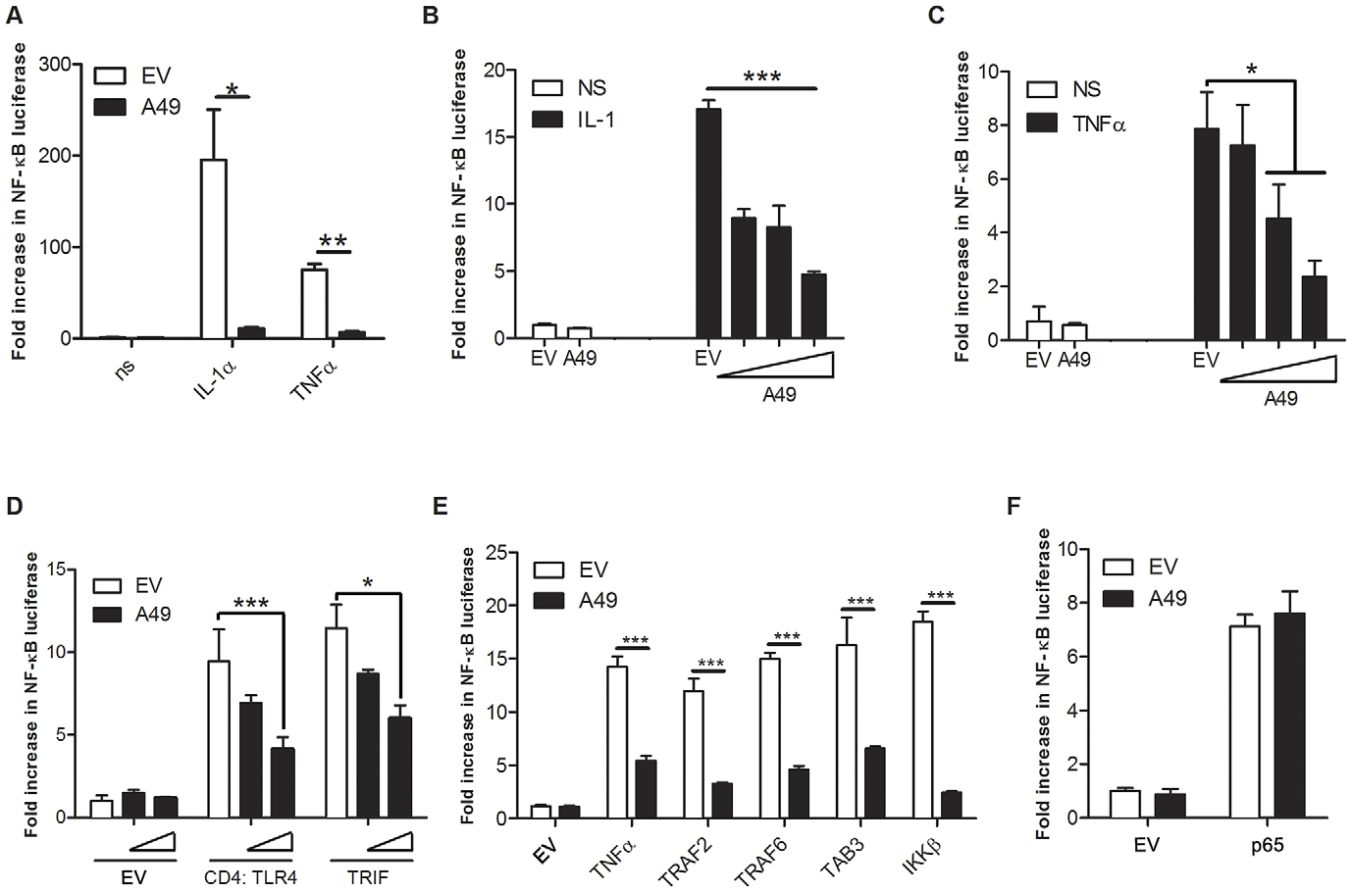
A49 is an NF-κB inhibitor. (A–F) Activation of firefly luciferase from an NF-κB-dependent promoter (NF-κB-Luc). Cells were transfected with NF-κB-Luc, a renilla luciferase control and an A49-expressing vector or empty vector (EV) for 24 h. When other proteins were co-expressed, these were transfected with the reporters and A49. Cells were stimulated 24 h later. NS, non-stimulated. (A) HeLa cells were stimulated with IL-1α or TNFα (100 ng/ml) for 6 h. (B–C) HEK293T cells were transfected with 50, 100 or 150 ng of pCMV-HA-A49 and then stimulated with (B) IL-1α (100 ng/ml) or (C) TNFα (250 ng/ml). (D–F) HEK293T cells were co-transfected with (D) CD4-TLR4 or TRIF, with (E) TRAF2, TRAF6, TAB3 or IKKβ, or with (F) p65 expression vectors. In all assays, data are presented as mean ± SD and show one representative experiment of at least three, each performed in triplicate. *p<0.05, **p<0.01 or ***p<0.001 comparing A49 transfected cells with EV.

To understand how A49 inhibited IFNβ promoter activity, additional reporter gene assays were performed. HEK293 cells were transfected with an NF-κB luciferase reporter and a plasmid expressing A49. Upon stimulation with either IL-1α or TNFα, A49 reduced NF-κB activation (Figure 3A) in a dose-dependent manner (Figure 3B, C). Moreover, A49 blocked NF-κB activation mediated by TLR signalling in HEK293T cells transfected with TLR4 fused to the CD4 dimerisation domain (CD4-TLR4) (Figure 3D). To determine where A49 was acting, NF-κB was activated by overexpression of proteins operating at different stages in the signalling cascade. A49 blocked NF-κB activation after overexpression of TRIF (Figure 3D), TRAF2, TRAF6, TGFβ-activated kinase 1 (TAK1)-binding protein 3 (TAB3), and IKKβ (Figure 3E). However, when p65 was overexpressed, A49 was not inhibitory (Figure 3F), showing that A49 suppresses NF-κB activation downstream of IKKβ and upstream of p65.

To test if A49 blocked other transcription factors, such as IRF3, HEK293ET cells were transfected with a ISG56.1 promoter reporter (for IRF3) [51], along with plasmids expressing VACV A49, B14, which blocks NF-κB activation [29], or C6, which blocks IRF3 activation [37]. Under the conditions tested, after stimulation with poly(I∶C), C6 blocked ISG56.1 activation, whereas A49 and B14 did not (Figure S3A). Similarly, A49 and B14 did not inhibit induction of the canonical ISRE reporter after poly(I∶C) stimulation, whereas C6 did (Figure S3B), and A49 did not inhibit the ISRE promoter after stimulation with IFNα (Figure S3C). Collectively, these results indicate that A49 inhibits NF-κB.

### A49 interacts with the ubiquitin E3 ligase β-TrCP

**Figure 4:**
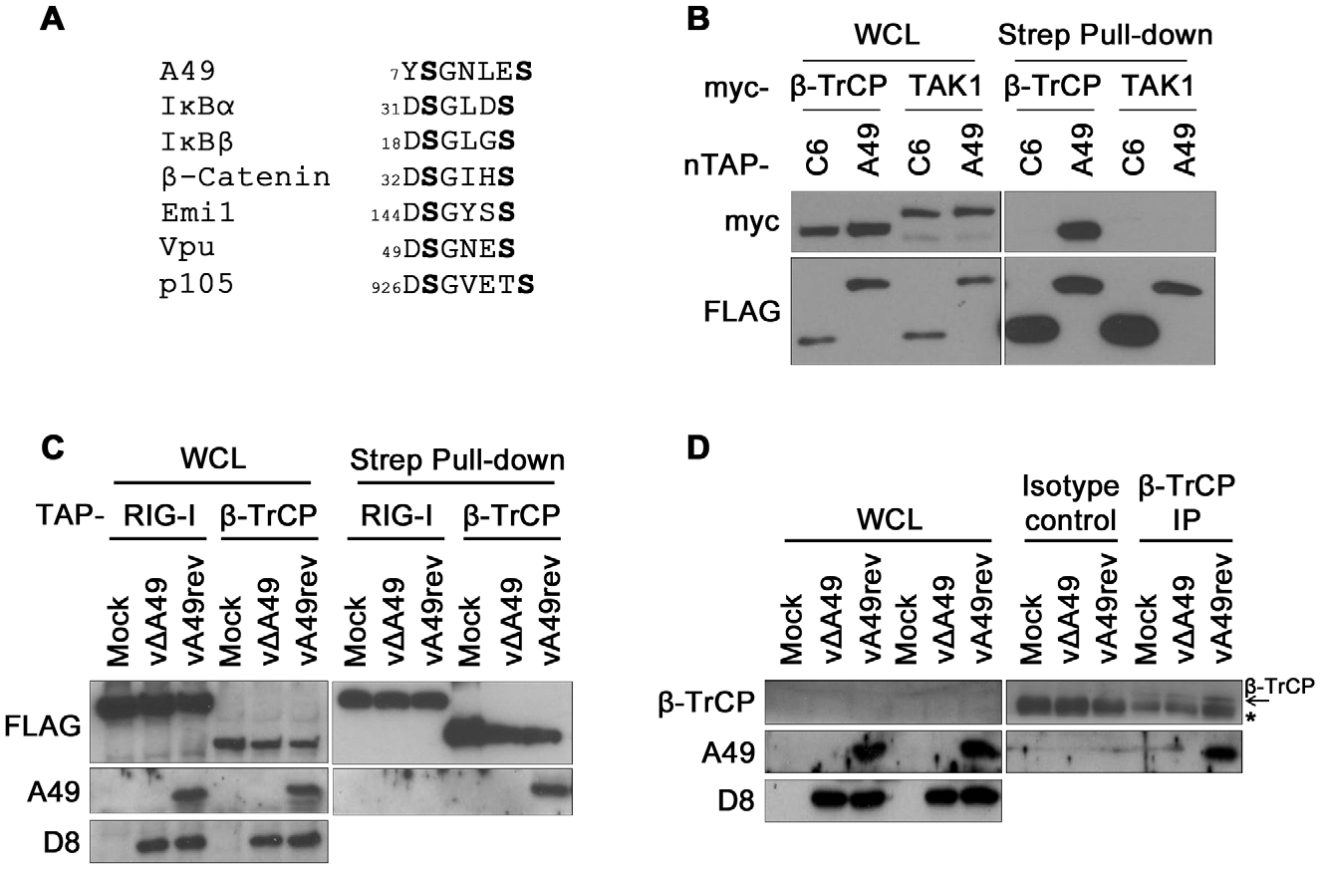
A49 interacts with the ubiquitin E3 ligase β-TrCP. (A) Alignment of β-TrCP recognition motif present in A49, IκBα, IκBβ, β-catenin, Emi1, HIV Vpu and p105. Phosphorylated serine residues are shown in bold and numbers indicate the first residue of the motif in each protein. (B) HeLa cells were cotransfected with myc-β-TrCP or myc-TAK1, with nTAP-A49 or nTAP-C6. Cells were lysed with IP buffer 24 h later and proteins pulled down with streptavidin beads. Whole cell lysate (WCL) (3%) of each sample was analysed. (C) HeLa cells were transfected with TAP-RIG-I or TAP-β-TrCP, and 24 h later were mock-infected or infected with vΔA49 or vA49rev at 10 PFU/cell for 6 h. Cells were lysed in IP buffer and a streptavidin pull-down was performed. D8 immunoblotting served as control for viral infection. WCL (1.5%) of each sample was analysed. (D) HeLa cells were either mock-infected or infected with vΔA49 or vA49rev with 10 PFU/cell for 6 h. After lysis with IP buffer, lysates were immunoprecipitated with a goat anti-β-TrCP antibody or an isotype control. WCL (1%) of each sample was analysed. Asterisk marks the antibody heavy chain. Data shown in (B) and (C) are one representative experiment of at least three. Data shown in (D) are one representative experiment out of two showing indistinguishable results.

Activation of NF-κB requires phosphorylation of IκBα on serines 32 and 36 within a short motif (DSGX_2–3_S) that is present in several proteins such as IκBα [8], Emi1 [52], β-catenin [12], HIV Vpu [11] and p105 [53]. Once this motif is phosphorylated, it is recognized by the E3 ligase β-TrCP [4]. Inspection of the A49 sequence identified the sequence SGNLES (aa 7–12) near the N terminus that matched the motif in β-TrCP substrates (Figure 4A). This suggested that A49 might bind β-TrCP and hence prevent β-TrCP from targeting its usual substrates. To test whether A49 interacted with β-TrCP, myc-tagged β-TrCP or TAK1 were co-expressed in HeLa cells with tandem-affinity purification (TAP)-tagged (streptavidin and FLAG) VACV proteins A49 or C6. After pull-down with streptavidin beads, an interaction between β-TrCP and A49 was seen by immunoblotting with anti-myc mAb (Figure 4B). Note that A49 did not interact with TAK1, nor did C6 with β-TrCP, confirming the specificity of the A49-β-TrCP interaction.

To test if this interaction occurred during virus infection, co-immunoprecipitation was done with extracts of HeLa cells transfected with TAP-tagged β-TrCP or retinoic acid induced gene I (RIG-I), and subsequently infected with vΔA49 or vA49rev. Both TAP-β-TrCP and TAP-RIG-I were pulled down with streptavidin beads and immunoblotting of the eluates showed that A49 associated only with TAP-β-TrCP (Figure 4C). Importantly, the β-TrCP-A49 interaction was demonstrated by immunoprecipitation of both proteins at endogenous levels after viral infection (Figure 4D).

### A49 binds the β-TrCP WD40 domain

**Figure 5:**
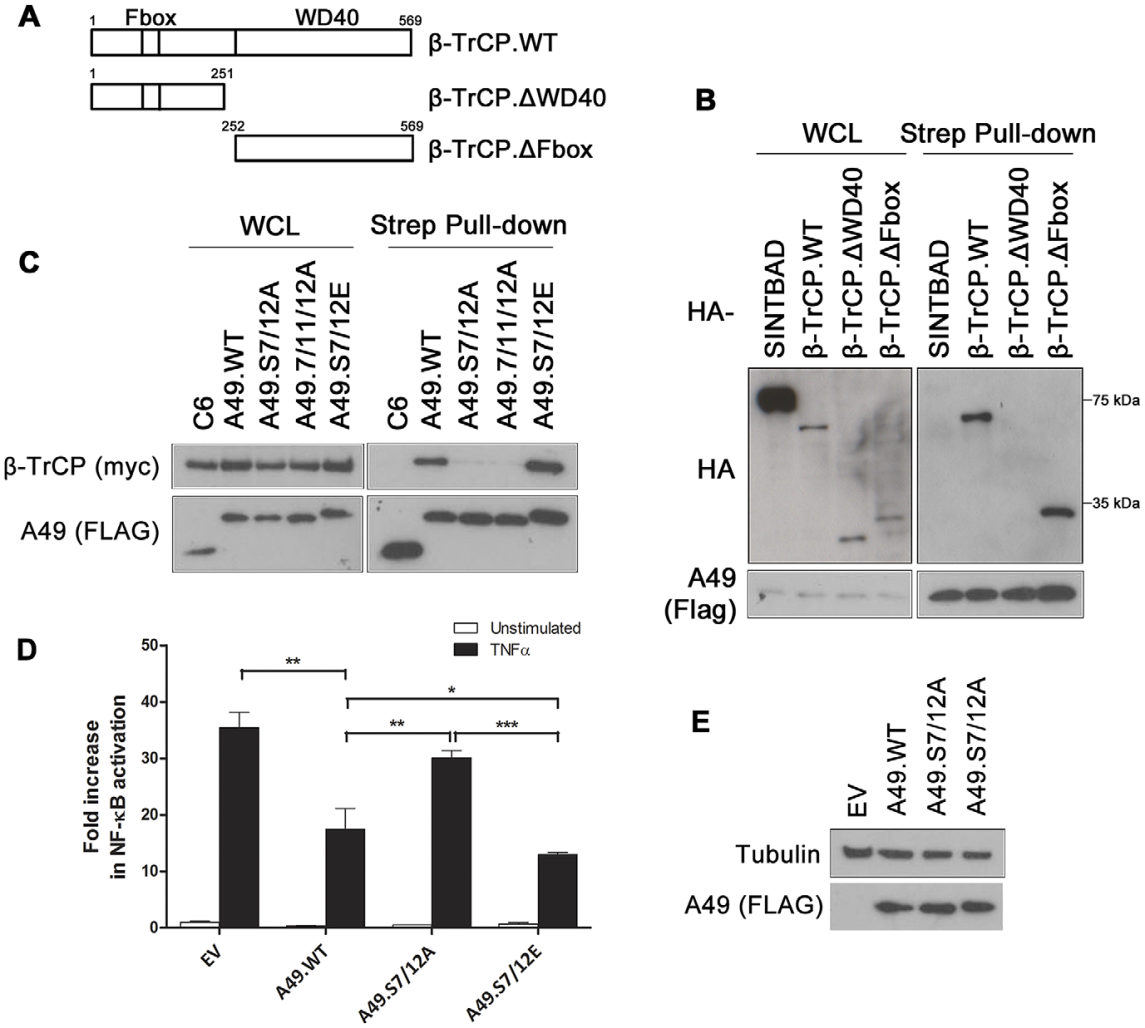
A49 binding to β-TrCP WD40 domain requires its N-terminal region. (A) Domains of β-TrCP and truncations generated. β-TrCP.ΔWD40 (1–250), β-TrCP.ΔF box (251–569). (B) HeLa cells were cotransfected with nTAP-A49 together with HA-tagged WT β-TrCP, ΔWD40 β-TrCP, ΔF-box β-TrCP or SINTBAD. After 24 h cells were lysed in IP buffer and a streptavidin pull-down was performed. WCL (2.5%) of each sample was analysed. (C) HeLa cells were cotransfected with myc-tagged β-TrCP together with nTAP-tagged WT A49, S7/12A A49, 7/11/12A A49, S7/12E A49 or C6 as a negative control. After 24 h cells were lysed in IP buffer and a streptavidin pull-down was performed. A fraction (2.5%) of each whole cell lysate (WCL) was analysed. (D) HEK293T cells were transfected with an NF-κB luciferase reporter, a renilla luciferase reporter and 100 ng of either nTAP-tagged WT, S7/12A or S7/12E A49 plasmids, or empty vector (EV). After 24 h cells were stimulated for 6 h with 20 ng/ml TNFα and luciferase activity was measured. Data are presented as mean ± SD and show one representative experiment of at least three, each performed in triplicate. *p<0.05, **p<0.01 or ***p<0.001 comparing conditions pair-wise as indicated. (E) Lysates from TNFα-stimulated samples from the reporter gene assays were fractionated and immunoblotted for FLAG and tubulin. WCL (10%) of each sample was analysed. Data shown in (B–C) are one representative experiment of at least three showing indistinguishable results.

The E3 ligase β-TrCP belongs to the F-box protein family and contains an N-terminal F-box domain and a C-terminal WD40 domain [9]. To study the A49-β-TrCP interaction, the F-box or the WD40 domains were deleted separately from β-TrCP (Figure 5A) and these truncated alleles were expressed in HeLa cells with TAP-tagged A49. After A49 pull-down, β-TrCP was co-purified only if it contained the WD40 domain (Figure 5B). This showed that A49, like IκBα, interacted with β-TrCP only if the WD40 domain was present, and that the F-box was dispensable.

To investigate if the IκBα-like motif within A49 was necessary for association with β-TrCP, A49 serines 7 and 12 were mutated to alanine (S7/12A) or glutamic acid (S7/12E). A third mutant was also made in which the glutamic acid at position 11 was changed to alanine in addition to serines 7 and 12 (7/11/12A). Expression of these TAP-tagged A49 alleles together with myc-tagged β-TrCP showed that the A49-β-TrCP interaction was lost with S7/12A or 7/11/12A, but was increased by the phospho-mimetic mutant S7/12E (Figure 5C). The association of A49 with another component of the β-TrCP SCF machinery that is found in complex with β-TrCP was also analysed. Both WT and S7/12E A49 co-immunoprecipitated with Skp1, but this was lost for the S7/12A A49 mutant (Figure S4). This is consistent with the SGLNES sequence near the A49 N terminus mediating binding to β-TrCP and, via β-TrCP, to other components of the SCF machinery.

Next, we addressed whether the IκBα-like motif within A49, which was required for β-TrCP interaction, was necessary for A49-mediated inhibition of NF-κB activation. HEK293T cells were transfected with an NF-κB responsive reporter plasmid together with TAP-tagged A49 alleles, and these cells were stimulated with TNFα. WT A49 inhibited NF-κB-luciferase expression compared to empty vector as expected, but the S7/12A mutant showed a statistically significant loss of function compared with WT (Figure 5D). It was also notable that the phospho-mimetic allele (S7/12E) inhibited NF-κB activation slightly more efficiently than WT A49 and this was consistent with its ability to bind β-TrCP slightly more strongly than WT (Figure 5D). Immunoblotting of these cell lysates revealed similar expression levels for each A49 allele (Figure 5E). Therefore, inhibition of NF-κB by A49 requires its β-TrCP recognition motif.

To compare the potency of A49 with another NF-κB inhibitor acting at the same stage of the pathway, a plasmid encoding the HIV Vpu protein fused at the N terminus with a TAP tag was transfected into HEK293 cells in parallel with TAP-tagged A49. Upon stimulation with TNFα or IL-1β, both A49 and Vpu inhibited NF-κB activation in a dose-dependent manner to a similar extent (Figure S5A, B). Immunoblotting of the lysates with a FLAG antibody demonstrated dose-dependent expression of each protein but HIV Vpu was expressed at higher levels than A49 suggesting that at equivalent levels of protein A49 was the more effective inhibitor under the conditions tested.

### A49 stabilises the IκBα/NF-κB complex and holds p65 in cytoplasm

**Figure 6:**
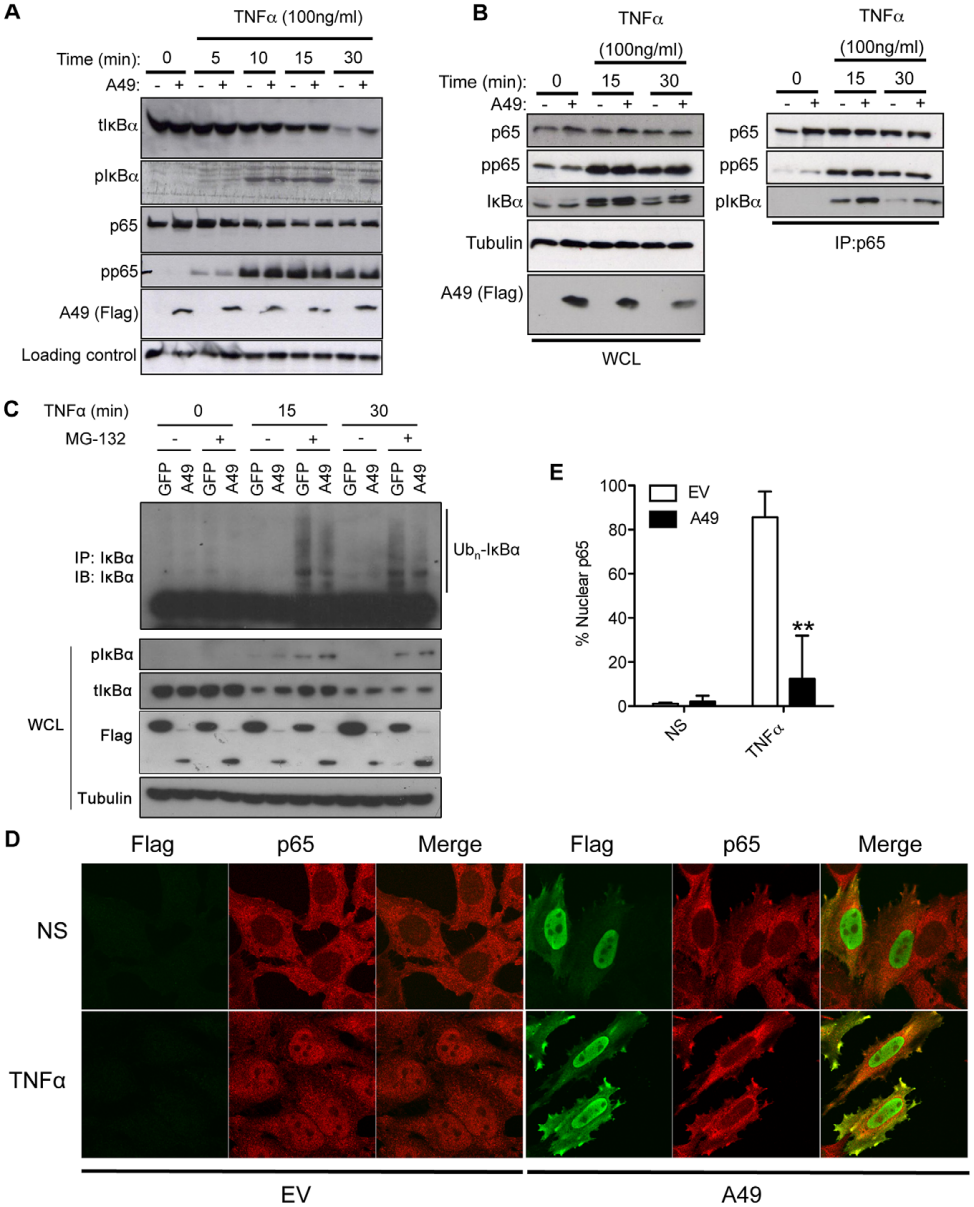
A49 stabilises the IκBα/NF-κB complex. (A) HEK293T cells were transfected with 10 µg of pCI-A49 (+) or empty vector (−). Cells were stimulated with TNFα (100 ng/ml) as indicated. Whole cell lysate (4%) of each sample was separated by SDS-PAGE and analysed by immunoblotting with the indicated antibodies indicated. (B) HEK293T cells were transfected as described in (A) and after stimulation with TNFα (100 ng/ml), lysed in IP buffer and lysates immunoprecipitated with anti-p65 antibody. WCL (2.5%) of each sample was loaded. (C) HEK293T cells were transfected with Flag-tagged A49 or Flag-tagged GFP as control. After 24 h cells were treated for 4 h with MG132 (25 µM) or vehicle only, and then stimulated with TNFα (200 ng/ml) as indicated. Cells were then lysed in IP buffer and lysates immunoprecipitated with anti-IκBα antibody. WCL (2%) of each sample was analysed. (D) HeLa cells on coverslips were transfected with pCI-A49 (A49) or empty vector (EV) and 24 h later were incubated with TNFα as indicated for 30 min. Cells were then stained for Flag (green) and p65 (red) and analysed by immunofluorescence. (E) The percentage of cells with nuclear stain for p65 was calculated (n = >100 for each sample). Each condition was performed in triplicates and the assay repeated twice. **p<0.01 comparing A49 transfected cells with the EV. Data shown in (A–C) are one representative experiment of at least three.

A49, like IκBα, contains a double serine motif that is needed to bind the C-terminal WD40 domain of β-TrCP. Therefore, it was possible that the ubiquitination and degradation of IκBα might be decreased in the presence of A49. As such, the IκBα/NF-κB complex would be stabilised and p65 would be retained in the cytoplasm. To address these possibilities, the effect of A49 expression on the level of total IκBα and p-IκBα (Ser 32/36) was examined by immunoblotting following TNFα stimulation (Figure 6A). In the absence of A49, p-IκBα was observed from 5 to 15 min after addition of TNFα and disappeared after 30 min, correlating with the reduction of IκBα levels at 30 mins. However, in the presence of A49, levels of both total IκBα and p-IκBα were higher when compared with cells transfected with the empty vector (Figure 6A). Conversely, A49 did not affect the phosphorylation of p65 on serine 536 by upstream kinases (Figure 6A), an event not involved in β-TrCP recognition. Furthermore, immunoprecipitation of p65 from HEK293T cells and immunoblotting for p-IκBα showed that the p-IκBα/p65 complex was de-stabilised by TNFα stimulation, but in the presence of A49 the complex remained intact (Figure 6B). To quantitate these effects, the intensity of the bands corresponding to p-IκBα and IκBα was analysed by densitometry. In all cases, the presence of A49 sustained both total and p-IκBα forms (Figure S6A, B).

Next the ubiquitination of IκBα was analysed in the presence and absence of A49. HEK293T cells expressing FLAG-tagged A49, or FLAG-tagged GFP, were treated with the proteasome inhibitor MG132 for 4 h and, following TNFα stimulation, IκBα was immunoprecipitated. After MG132 treatment, ubiquitinated forms of IκBα were observed upon TNFα activation, but these were reduced in the presence of A49 (Figure 6C). Densitometry analysis of the intensity of the higher molecular mass forms corresponding to ubiquitinated IκBα confirmed a reduction in IκBα ubiquitination in the presence of A49 (Figure S6C). To characterise the effects of A49 further, the levels of p27, a known target of the F-box protein Skp2, were measured in the presence of A49 and MG132. p27 accumulated after inhibition of the proteasome, but was unaffected by A49, suggesting that A49 did not interfere with the processing of targets of other F-box proteins or affected the proteasome non-specifically (Figure S7).

The translocation of p65 into the nucleus upon TNFα stimulation in the presence or absence of A49 was analysed. In HeLa cells A49 was present in both the nucleus and cytoplasm before and after treatment with TNFα and prevented the TNFα-induced translocation of p65 into the nucleus (Figure 6D). Quantitation showed highly significant differences (Figure 6E).

Collectively, these results show that A49 binds β-TrCP and thereby diminishes ubiquitination of p-IκBα. This stabilises p-IκBα and its interaction with NF-κB, so retaining p65 in the cytoplasm and preventing NF-κB-dependent gene expression.

### A49 interferes with IκBα phosphorylation and degradation during infection

**Figure 7:**
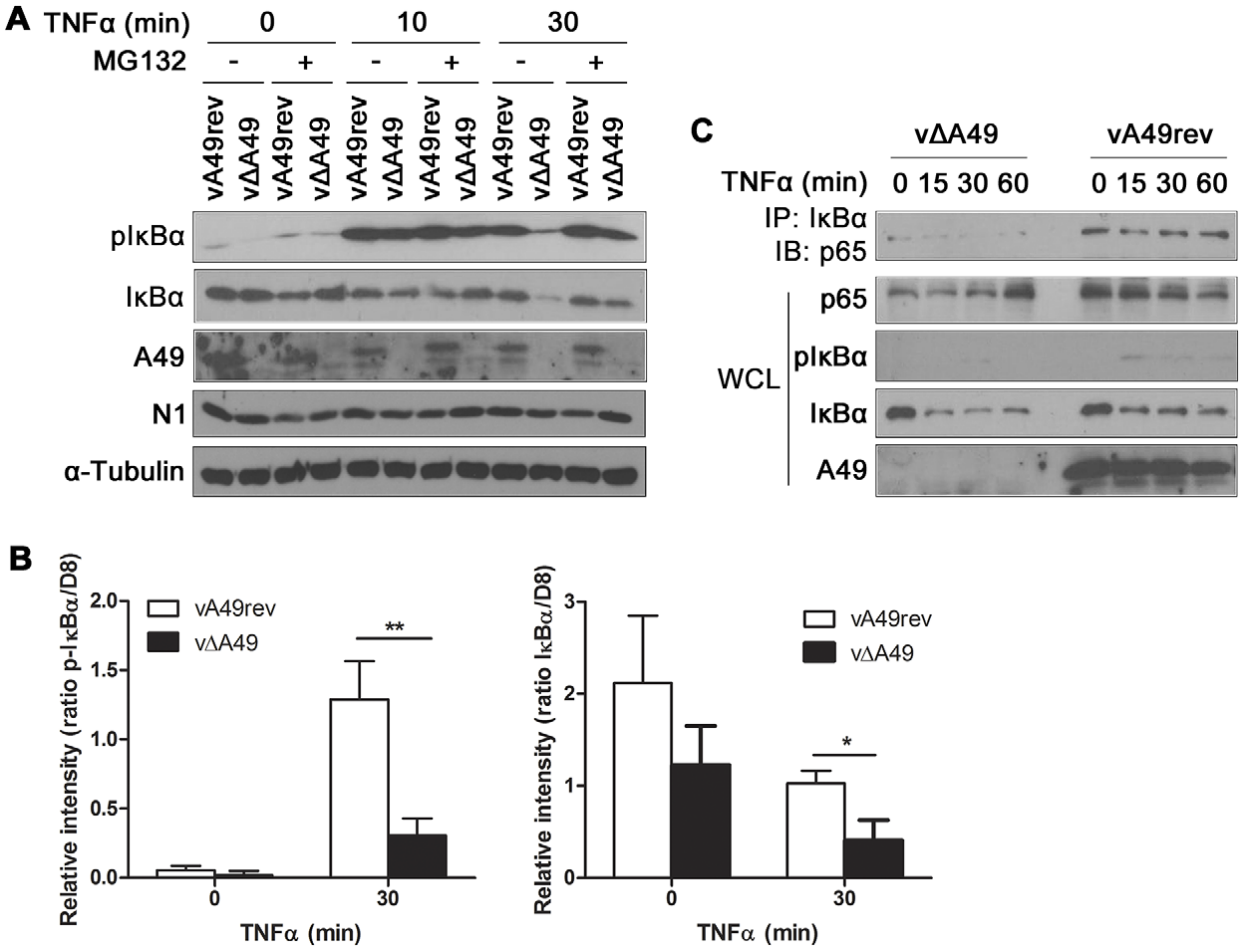
A49 interferes with IκBα degradation during viral infection. (A) HeLa cells were infected with vA49rev or vΔA49 at 10 PFU/cell for 4 h, treated for 1 h with MG132 (20 µM) or vehicle only, and then stimulated with TNFα (200 ng/ml) as indicated. Cell extracts were separated by SDS-PAGE and analysed by immunoblotting with the antibodies indicated. N1 immunoblotting served as control for viral infection. (B) HeLa cells were infected and treated with TNFα in triplicate as in (A) and cell extracts were analysed by quantitative fluorescence immunoblotting. The amounts of p-IκBα and IκBα are shown as ratios compared with VACV protein D8. *p<0.05 or **p<0.01 comparing vA49rev with vΔA49. (C) HeLa cells were infected with vA49rev or vΔA49 at 10 PFU/cell for 6 h and then stimulated with TNFα (200 ng/ml) as indicated. Cells were then lysed in IP buffer and the lysates were immunoprecipitated with anti-IκBα antibody and immunoblotted for p65. In each assay, 2% of WCL of each sample was immunoblotted with the indicated antibodies.

Next, A49 function was tested during VACV infection. HeLa cells were infected with vA49rev or vΔA49 for 4 h, treated with MG132 for 1 h, and then stimulated with TNFα for 10 or 30 min. Infection by a VACV expressing A49 prevented IκBα degradation and stabilised p-IκBα, whereas infection with vΔA49 did not (Figure 7A). Remarkably, vA49rev induced accumulation of p-IκBα even without TNF stimulation, indicating that A49 blocked NF-κB activation triggered by viral infection. In addition, failure to accumulate p-IκBα could be reversed by MG132 (both without TNF activation or 30 min post-activation), suggesting that no other VACV protein interfered with the proteasomal degradation of p-IκBα downstream of A49. To obtain a more quantitative read-out, a similar experiment in which cells were infected with vA49rev or vΔA49 and treated with TNFα for 30 mins, was performed in triplicate. The amounts of p-IκBα and total IκBα were determined by quantitative fluorescence imaging of immunoblots, and plotted as a ratio compared to the amount of viral protein D8 to account for the efficiency of infection. Infection with vA49rev sustained levels of both p-IκBα and total IκBα 30 mins post-treatment compared to infection with vΔA49, and these differences were statistically significant (Figure 7B). In the absence of TNFα, accumulation of p-IκBα and IκBα during vA49rev infection was also detected (as observed by conventional immunoblotting), but with the sample sizes tested this was not significant.

Lastly, the stability of the IκBα/NF-κB complex was assessed during viral infection. HeLa cells were infected with vΔA49 or vA49rev and total IκBα was immunoprecipitated after TNFα stimulation. vA49rev infection stabilised both total IκBα and p-IκBα and these remained associated with p65 (Figure 7C). In contrast, vΔA49 infection failed to inhibit IκBα degradation and consequently no p65 co-precipitated with IκBα. So although there are other NF-κB inhibitors expressed by vΔA49, the A49 protein seems dominant in stabilising the IκBα/NF-κB complex.

## Discussion

The IRFs and NF-κB transcription factors are central to a coordinated immune response and their activation culminates in the expression of IFNβ, which induces an antiviral state in cells, and the production of inflammatory cytokines and chemokines which recruit lymphoid cells to the site of infection. In response to this host defence, viruses have evolved many mechanisms to suppress the host immune response and complete their life cycle. Understanding how viruses evade immune responses can aid understanding of virus pathogenesis and of the immune system itself.

**Figure 8:**
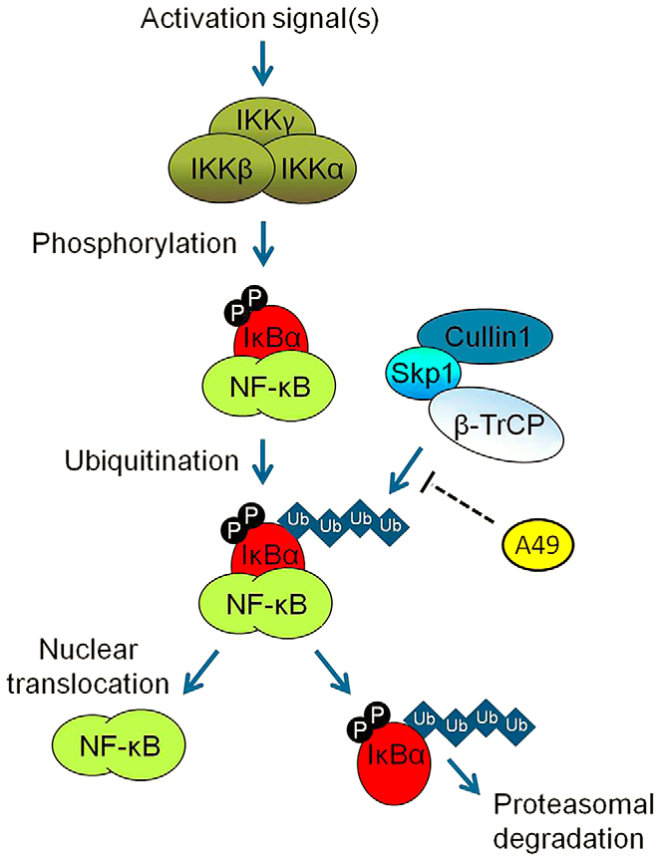
Inhibition of NF-κB activation by A49. Model depicting how poxvirus protein A49 interferes with activation of the transcription factor NF-κB by targeting the E3 ligase β-TrCP and thus preventing the degradation of IκBα.

Here, the VACV WR A49 protein is shown to be an inhibitor of innate immune signalling by blocking NF-κB activation. A49 was identified as an inhibitor of IFNβ expression in a screen of VACV proteins encoded near the genome termini, and dissection of the mechanism by which A49 inhibited IFNβ activation showed that A49 blocked NF-κB activation by binding to the WD40 domain of the E3 ubiquitin ligase β-TrCP (Figure 4 and 5). Thus, even though IκBα was phosphorylated by upstream kinases, β-TrCP-mediated ubiquitination of p-IκBα was reduced, p-IκBα was stabilised in complex with NF-κB and so NF-κB was retained in the cytosol (Figure 8).

A49 mediates its anti-NF-κB activity via molecular mimicry. Near the N terminus of A49 there is a SGLNES sequence that is closely related to motifs in IκBα, IκBβ, HIV Vpu and other β-TrCP substrates (Figure S1, 4A) suggesting that A49, like these other proteins, might bind β-TrCP. This interaction was shown by reciprocal immunoprecipitation of transfected tagged molecules and of endogenous β-TrCP and A49 made during VACV infection (Figure 4). Mutagenesis of the conserved serines within SGLNES to alanines prevented interaction with β-TrCP, stabilisation of p-IκBα, and inhibition of NF-κB activation (Figures 5 and S4). Conversely, mutation of these residues to glutamic acid increased the binding of A49 to β-TrCP and enhanced the inhibitory activity of A49 (Figure 5). This observation is in contrast to mutagenesis of IκBα where substitution of phospho-serines with glutamic or aspartic acid reduced IκBα recognition [8]. This indicates that in the A49 motif, charge-based interactions are sufficient to support binding. Notably, between the conserved serines A49 is one residue longer than in IκBα, like some β-TrCP substrates e.g. p105 [53]. This residue is after the glycine and before the hydrophobic amino acid known to insert in the β-TrCP hydrophobic groove [54].

The A49 protein is conserved in the majority of VACV strains and orthopoxviruses (OPVs) including variola virus, the causative agent of smallpox (Figure S1). But in ectromelia, camelpox and monkeypox viruses the A49 coding region is disrupted by mutation, showing A49 was non-essential for replication of those OPVs. The isolation of vΔA49 which replicated normally in cell culture and produced a normal sized plaque confirmed A49 is dispensable for VACV also (Figure S2). However, vΔA49 was less virulent compared to controls *in vivo* and this attenuation was characterised by lower weight loss, more rapid recovery and lower virus titres in infected lungs on days 5 and 7 pi (Figure 1). This attenuation, and the substantial increase of p-IκBα stability mediated by A49 during infection (Figure 7), was despite the fact that VACV expresses several other inhibitors of NF-κB activation (see introduction). The activity of A49 is clearly, therefore, not redundant. This might be explained by either these different proteins acting at different stages in the activation pathway, or them having multiple functions. In the former case, if an NF-κB inhibitor acted only downstream of the IL-1R or the TNFR before these pathways converge, it might give a different phenotype *in vivo* to an inhibitor that acted downstream of where these pathways converge. In addition, since there is crosstalk between the NF-κB pathway and other pathways, such as the MAP kinases for instance, an inhibitor might affect these other pathways too depending on its site of action. Concerning the possibility that the NF-κB inhibitors might have multiple functions, this has already been demonstrated in some cases. For instance, the N1 protein not only inhibits NF-κB activation [28], [47] but also inhibits apoptosis [55] and these functions are assigned to different binding surfaces of the protein [47]. With A49, there is a parallel with the HIV Vpu protein, which contains the conserved motif for interaction with β-TrCP, and has more than one function. Not only does Vpu interact with β-TrCP and so diminish degradation of p-IκBα, and hence block NF-κB activation [56], but Vpu also engages other proteins such as CD4 and tetherin and so brings these to β-TrCP for ubiquitination and degradation [57]. It is possible that A49 will also bind other cellular proteins and target these for β-TrCP-mediated ubiquitination and degradation in a manner advantageous for VACV. The identification of such targets may require their stabilisation by use of proteasomal inhibitors or mutants of A49, such as the S7/12A, which no longer bind to β-TrCP.

A comparison of the VACV-encoded inhibitors of NF-κB B14 and A49 is particularly interesting. The B14 protein binds IKKβ and reduces phosphorylation of IκBα, and thereby activation of NF-κB [24], [29]. In contrast, A49 reduces ubiquitination of p-IκBα and thus stabilises it. So B14 is acting at the step in the NF-κB activation pathway immediately upstream of the A49 protein. The benefit of multiple viral inhibitors of NF-κB was demonstrated here by the detection of more p-IκBα after infection with vA49rev than vΔA49 despite the presence of B14 in both viruses (Figure 7A, B). Therefore, these viral inhibitors work in combination to abrogate NF-κB activation during viral infection and each contributes to virus virulence. But, unexpectedly, the attenuation induced by loss of B14 or A49 is apparent in different *in vivo* models. In an intradermal model of infection [58], [59] a virus lacking B14 showed attenuation, but this virus had normal virulence in the intranasal model [25]. Conversely, vΔA49 was attenuated in the intranasal model (Figure 1) but not in the intradermal model (data not shown). This intriguing difference might be explained by either A49 or B14, or both proteins, having additional functions.

Although many inhibitors of NF-κB had been reported in VACV previously, genetic evidence had suggested the existence of additional inhibitor(s) because the VACV strain v811 stabilised p-IκBα despite lacking all known inhibitors of TNFα-mediated NF-κB activation [36]. The *A49R* gene is present in mutant v811 [60] and so the A49 protein probably represents such an inhibitor. A49 represents one of several virus proteins that target β-TrCP. In addition to A49 and HIV Vpu, rotavirus [61] and Epstein-Barr virus [62] also modulate β-TrCP activity. The widespread targeting of β-TrCP illustrates the importance of the SCF^β-TrCP^ complex for pathogen-induced responses.

In conclusion, the VACV A49 protein inhibits β-TrCP function by molecular mimicry and thereby blocks NF-κB activation, promotes immune evasion and enhances virus virulence. Given that a highly conserved version of A49 is encoded by all (∼50) strains of variola virus sequenced [63], it is probable that this strategy for increasing virulence by immune evasion is conserved in the pathogens that cause smallpox and AIDS.

## Materials and Methods

### Ethics statement

This work was carried out in accordance with regulations of The Animals (Scientific Procedures) Act 1986. All procedures were approved by the UK Home Office and carried out under the Home Office project licence PPL 70/7116.

### Expression vectors, antibodies and reagents

For mammalian expression, *A49R* was cloned into a pCI vector (Promega) with a Flag tag, into a pcDNA4/TO vector (Invitrogen) as an N-terminal TAP fusion containing 2 copies of the streptavidin binding sequence and 1 copy of the FLAG epitope [64], and into pCMV-HA (Clontech) with an N-terminal HA tag. Mutagenesis of A49 was performed by PCR amplification using forward primers containing the desired mutations. nTAP.C6 and FLAG-B14 have been described [29], [37]. To produce protein in bacteria (used for antibody generation), *A49R* was cloned into the pOPINE vector [65]. Myc-β-TrCP was obtained from Addgene (identified as β-TrCP2 after sequencing). The ORF was PCR amplified and cloned in pcDNA4/TO as TAP or HA fusions. PCR products covering residues 1–250 (F-box) or 251–569 (WD40) were cloned fused with HA. The fidelity of the PCR products was verified by DNA sequencing.

A polyclonal antibody against A49 was generated in rabbits by Eurogentec immunised with purified recombinant A49 protein. Monoclonal antibody against IκBα was a kind gift of Ron T. Hay (University of Dundee) and was used in ubiquitination assays. Other antibodies were: p65 (Santa Cruz), phospho-p65 (Ser 536) (Cell Signalling), IκBα and p-IκBα (Ser32/36) (Cell Signalling), β-TrCP (clone C-18, Santa Cruz), Skp1 (Santa Cruz), p27 (Cell Signalling), β-actin (Abcam), α-tubulin (Upstate Biotech), myc (Cell Signalling), HA (Covance) and FLAG (M2 clone, Sigma). The mouse monoclonal antibody AB1.1 against D8 was described [66] as well as the anti-N1 serum [45]. Poly(I∶C), poly(dA-dT) and MG132 were from Sigma, TNFα, IL-1β and IL-1α were from Peprotech, LPS and CpG were from Invitrogen. Sendai virus (strain Cantell) was grown in embryonated hen eggs [67] and was used at a single dose, at a dilution of 1∶200.

### Cell culture

BSC-1, CV-1, HEK293T, HEK293ET and RAW 264.7 cells were cultured in Dulbecco's modified Eagle's medium (DMEM, Gibco) supplemented with 10% heat-treated foetal bovine serum (FBS, Harlan Sera-Lab), 50 IU/ml penicillin and 50 µg/ml streptomycin (Gibco) and 2 mM L-glutamine (Gibco). HeLa cells were maintained in Minimum Essential Medium (MEM - Gibco) supplemented with 1× non-essential amino acid solution (Sigma) and 10% heat-treated (56°C, 1 h) foetal bovine serum (FBS, Harlan Sera-Lab), 50 IU/ml penicillin and 50 µg/ml streptomycin (Gibco) and 2 mM L-glutamine (Gibco).

### Bioinformatics

Alignment of the A49 amino acid sequence from poxviruses was performed using Clustal X and Genedoc [68]. Viruses and GenBank accession numbers are: VACV-Cop (vaccinia virus strain Copenhagen, acc. num. M35027), VACV-WR (vaccinia virus strain Western Reserve, AY243312), VACV-TT (vaccinia virus strain Tian-Tian, AF095689), CPXV (cowpox virus, NC_003663), HSPV (horsepox virus, DQ792504), VARV (variola virus strain India 1967, NC_001611).

### Generation of recombinant viruses

*A49R* gene fragments were produced by PCR using VACV WR genomic DNA as template and were cloned into a pCI (Promega) derived plasmid. This plasmid contained *E. coli* guanylphosphoribosyl transferase (*EcoGPT*) fused in frame with the enhanced green fluorescent protein (*EGFP*), driven by a VACV promoter and enables transient dominant selection of recombinant viruses [69]. To produce an A49 deletion VACV, a DNA fragment containing the left and right flanking regions of *A49R* was produced by overlapping PCR. The 5′ fragment was generated with oligonucleotides 5′- CAGGGATCCAACAAAAGGTATTACAAGAAT – 3′ (LA), containing a *Bam*HI restriction site (underlined), and 5′- *ATATCGTTCGCGGAT*ATAGTTTCTATCTTGGCAATAAC 3′ containing nucleotides from the 3′ fragment (italics) at the 5′ end. The 3′ fragment was generated with oligonucleotides 5′- *CAAGATAGAAACTAT*ATCCGCGAACGATATTTGTG -3′, with complementary sequence to the 5′ fragment (italics) and 5′- TGCAGCGGCCGCCGGATTTCTGTGTTCTCTTTGAAG -3′ (RA), containing a *Not*I restriction site. These two fragments were joined by PCR using the LA and RA oligonucleotides and cloned forming pΔA49. To make vΔA49, BSC-1 cells were infected with VACV WR and transfected with pΔA49. Recombinant viruses were collected 24 h later selected in the presence of mycophenolic acid, xanthine and hypoxanthine [69]. Intermediate EcoGPT^+^ viruses were resolved into WT or vΔA49 by plaquing on BSC-1 cells in the absence of drugs and their genotype confirmed by PCR. To generate pA49rev, a DNA fragment containing the entire *A49R* gene and flanking regions was generated with oligonucleotides LA and RA. vA49rev was generated in a similar manner by transfection of pA49rev in vΔA49-infected cells.

### Virus growth curves

Growth kinetics of viruses was determined as described [37].

### Plaque size assay

Virus plaque size was determined as described [70].

### *In vivo* experiments

Virus virulence was analysed in a murine intranasal infection model [71]. Groups of 5 BALB/c mice 6–8 weeks old were inoculated with 5×10^3^ PFU of the different recombinant virus in 20 µL PBS. Mice were weighed daily and signs of illness were recorded as described [43]. All experiments were conducted at least twice.

### Reporter assays

HEK293T cells in 96-well plates were transfected with 60 ng/well of firefly luciferase reporter plasmids, 10 ng/well of pTK-Renilla luciferase (pRL-TK, Promega) or 20 ng/well of pGL3-renilla luciferase [37] as transfection control, and the indicated amount of expression vectors with FugeneHD (Roche) or GeneJuice (Merck). A plasmid encoding HIV Vpu was a gift from Paul Lehner, and was amplified by PCR and cloned as N-terminal TAP fusion. IFNβ-promoter luciferase reporter was a gift from T. Taniguchi (University of Tokyo, Japan) and NF-κB-luciferase was from R. Hofmeister (University of Regensburg, Germany). ISRE-luciferase and pRL-TK (Renilla Luciferase) were purchased from Promega. ISG56.1 was a gift from Ganes Sen (Cleveland Clinic, USA). TLR3 was a gift from D.T. Golenbock (University of Massachusetts Medical School, USA). The concentration of the A49 expression vectors varied according to the vector used and the experiment. They are: for Figures 2A, B and Figures 3A, E and F, 60 ng/well; Figure 2D, 50 and 150 ng/well; Figures 3B, 3C and S5, 50, 100 or 150 ng/well; Figure 3D, 150 or 50 ng/well. DNA was kept constant during the transfections by the addition of empty vector control plasmid. Cells were stimulated as indicated in the figures and were harvested in passive lysis buffer (Promega). The relative stimulation of reporter-gene expression was calculated by normalizing firefly luciferase activity with renilla luciferase activity. In all cases, data shown are representative from at least three independent experiments. Data from experiments performed in triplicate are expressed as mean ± SD.

### Real-time PCR

RNA from HEK293T cells in 6-well plates was extracted using the RNeasy kit (QIAGEN) and converted to cDNA using the Quantitect RT kit (QIAGEN). IFNβ mRNA was quantified by real-time PCR with the TaqMan gene expression assay Hs00277188_s1 and a β-actin endogenous control VIC-MGB probe (6-carboxyrhodamine–minor groove binder; Applied Biosystems). Cells were transfected with 2 µg/well of the A49 expression plasmid or the empty vector. Experiments were performed in triplicate.

### ELISA

HEK293T cells were stimulated as indicated in figure legends. Supernatants were analysed for CCL5 protein using Duoset reagents (R&D Biosystems).

### Co-immunoprecipitation

For co-immunoprecipitation, HEK293T or HeLa cells were transfected using Fugene-6 (Roche). After 24 h, cells were washed once with ice-cold PBS and lysed with IP buffer (10% glycerol, 150 mM NaCl, 20 mM Tris-HCl [pH 7.4], 0.1% Triton-X100, and protease inhibitors [Roche]). Lysates were incubated with either streptavidin beads (Thermo Scientific) or protein G sepharose beads (GE Life Sciences) that were pre-incubated with the corresponding antibody for 2 h at 4°C. After 3 washes with ice-cold Tris-buffered saline (25 mM Tris-HCl, pH 7.4, 150 mM NaCl, 2 mM KCl), proteins were eluted and analysed by SDS-PAGE and immunoblotting.

### Densitometry and quantitative fluorescence immunoblotting

Densitometry analysis was performed using ImageJ, an open-source image processing and analysis software provided by the National Institutes of Health (http://rps.info.nih.gov/ij). Films were transformed into digital pictures and intensities were calculated after removal of background signal. For quantitative immunoblotting, IRDye 800-conjugated donkey anti-mouse and goat anti-rabbit antibodies were used according to the manufacturer's instructions (LI-COR Biosciences). Membranes were then dried and scanned using the Odyssey infrared imaging system (LI-COR Biosciences). Quantitation was performed using the system software to determine total band intensities on the original scans.

### Immunofluorescence

HeLa cells on glass coverslips were transfected (Fugene 6) with 50 ng of plasmid expressing A49. After 24 h, cells were either treated with 50 ng/ml TNFα (Peprotech) (diluted in warm 2% FBS MEM) or were mock treated. After 30 min, the cells were stained with anti-p65 (1∶50) and anti-FLAG (1∶500) and prepared for imaging as described [35].

### Statistical analysis

Data were analysed using unpaired Student's T test unless stated otherwise. Statistical significance is expressed as follows: * P-value<0.05, ** p-value<0.01, *** p-value<0.001.

## Supporting Information

Figure S1 ***A49R*** **is a non-essential gene for VACV replication.** (A) Alignment of the A49 amino acid sequence of VACV and some other orthopoxviruses. The boxed region defines the β-TrCP recognition region. (B) BSC-1 cells were infected with VACV strain WR at 5 PFU/cell in the presence or absence of AraC. Cells were harvested at the indicated times and lysates were prepared and analysed by SDS-PAGE and immunoblotting for tubulin and VACV proteins D8 and A49, as indicated. Whole cell lysate (5%) of each sample was loaded.
(TIF)

Figure S2 ***A49R*** **is a non-essential gene for VACV replication.** (A–B) CV-1 cells were infected with vA49WT, vΔA49 or vA49rev at (A) 0.01 PFU/cell or (B) 10 PFU/cell. Cells were harvested at the indicated times (A) and 24 h post-infection (B), and infectious VACV was titrated by plaque assay on BSC-1 cells monolayers. (C) BSC-1 cells were infected with vA49WT, vΔA49 or vA49rev at 0.01 PFU/cell and after 72 h the monolayer was stained with crystal violet and the plaque diameter measured using Axiovision 4.6 software and a Zeiss Axiovert 200 M microscope. Results are expressed as the mean plaque radius ± SD.
(TIF)

Figure S3 **A49 does not inhibit ISRE or IRF3 activation.** (A–B) HEK293ET cells were transfected with empty vector (EV), B14, C6 or A49, together with a renilla luciferase and (A) the IRF3-specific reporter ISG56.1-Luc or (B) an ISRE-Luc. After 24 h cells were stimulated with poly(I∶C) for 6 h and the luciferase activity was measured. (C) HeLa cells were transfected with pCI-A49 or empty plasmid (EV), an ISRE-Luc reporter and the TK-renilla control. After 24 h cells were treated with 500 u/ml of IFNα for 6 h and the luciferase activity was measured. Data are presented as mean ± SD and show one representative experiment of at least three, each performed in triplicate. *p<0.05 or ** p<0.01 comparing A49, C6 or B14 transfected cells with EV.
(TIF)

Figure S4 **A49 binds the SCF machinery via β-TrCP.** HeLa cells were transfected with plasmids encoding WT A49, or mutants S7/12A A49 or S7/12E A49, or VACV protein C6. Each VACV protein was fused at the N-terminus with a TAP tag. After 24 h, cells were lysed in IP buffer and a streptavidin pull-down was performed. Samples were analysed by SDS-PAGE and immunoblotting with the indicated antibodies. Whole cell lysate (WCL, 2%) of each sample was loaded.
(TIF)

Figure S5 **Inhibition of NF-κB activation by A49 and Vpu.** (A–B) HEK293T cells were transfected with empty vector (EV), of plasmids expressing A49 or Vpu, together with a renilla luciferase and the NF-κB-Luc reporter. After 24 h cells were stimulated with TNFα (A) or IL-1β (B) for 6 h and the luciferase activity was measured. Whole cell lysate (12.5%) of each sample was analysed by SDS-PAGE and immunoblotted for FLAG and actin. Data are presented as mean ± SD and show one representative experiment of at least three, each performed in triplicate. *p<0.05 or ** p<0.01 comparing A49 or Vpu transfected cells with EV.
(TIF)

Figure S6 **Quantitation of p-IκBα, IκBα, and ubiquitinated IκBα, by densitometry.** Intensity of the bands corresponding to p-IκBα, IκBα (A–B) and ubiquitinated IκBα C) from pictures shown in Figure 6 was analysed by ImageJ and represented as bars in its arbitrary units (AU) after subtracting background signal.
(TIF)

Figure S7 **A49 does not affect the proteasome non-specifically.** HeLa cells were transfected with pCI-A49 (A49) or the empty vector and, 24 h later, were treated with MG132 (20 µM) for different lengths of time. Cell extracts (4% of total) were prepared separated by SDS-PAGE and analysed by immunoblotting with the antibodies indicated.
(TIF)
