# Supplementary figures and images for: Poxvirus Targeting of E3 Ligase β-TrCP by Molecular Mimicry: A Mechanism to Inhibit NF-κB Activation and Promote Immune Evasion and Virulence
Source: PLoS Pathog. 2013 Feb 28;9(2):e1003183. doi: 10.1371/journal.ppat.1003183 (PMC3585151; doi:10.1371/journal.ppat.1003183)

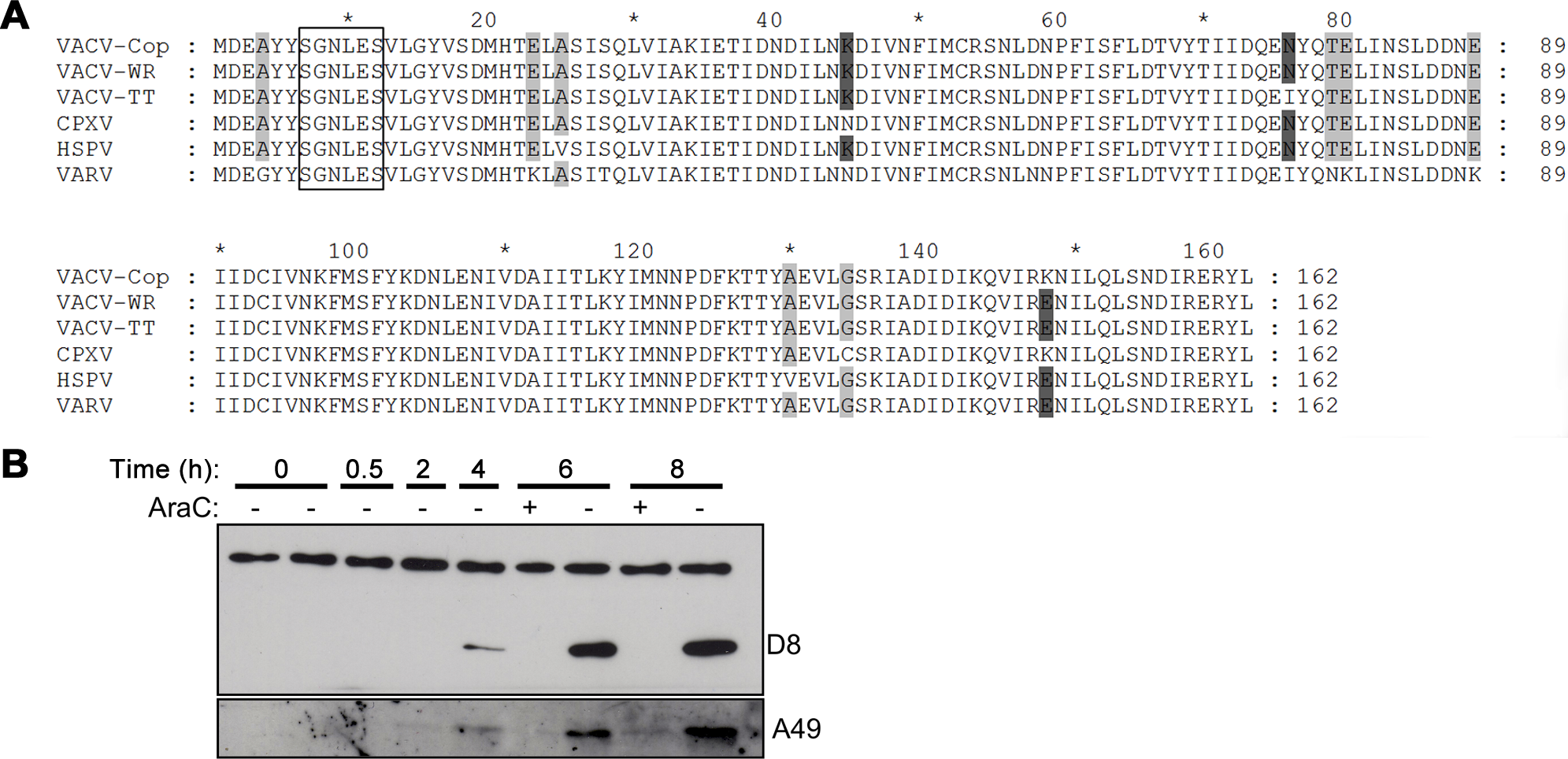

Supplement: Figure S1 — A49R is a non-essential gene for VACV replication. (A) Alignment of the A49 amino acid sequence of VACV and some other orthopoxviruses. The boxed region defines the β-TrCP recognition region. (B) BSC-1 cells were infected with VACV strain WR at 5 PFU/cell in the presence or absence of AraC. Cells were harvested at the indicated times and lysates were prepared and analysed by SDS-PAGE and immunoblotting for tubulin and VACV proteins D8 and A49, as indicated. Whole cell lysate (5%) of each sample was loaded. (TIF) [file ppat.1003183.s001.tif]

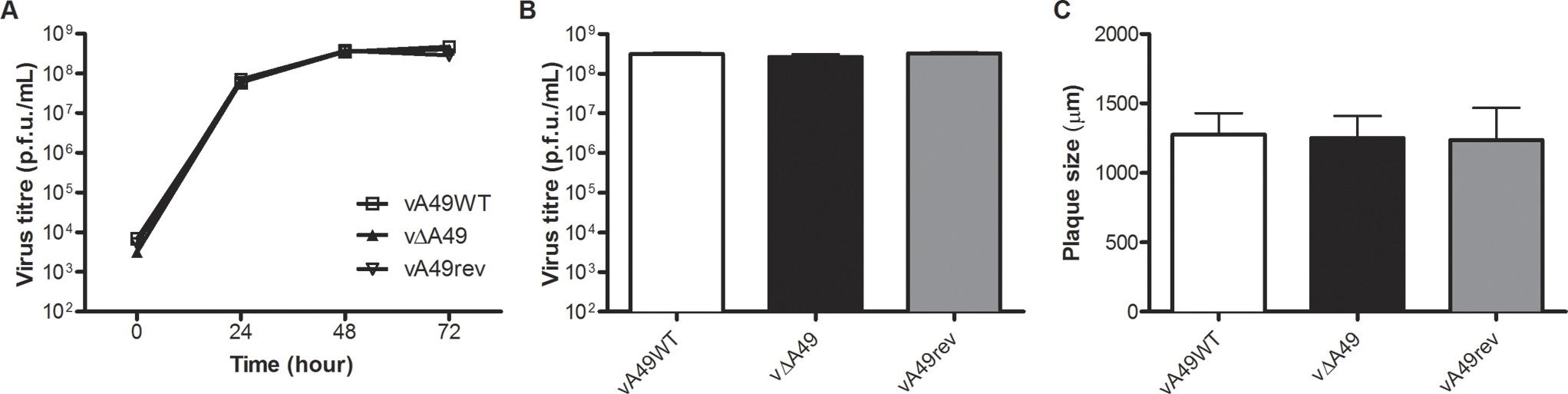

Supplement: Figure S2 — A49R is a non-essential gene for VACV replication. (A–B) CV-1 cells were infected with vA49WT, vΔA49 or vA49rev at (A) 0.01 PFU/cell or (B) 10 PFU/cell. Cells were harvested at the indicated times (A) and 24 h post-infection (B), and infectious VACV was titrated by plaque assay on BSC-1 cells monolayers. (C) BSC-1 cells were infected with vA49WT, vΔA49 or vA49rev at 0.01 PFU/cell and after 72 h the monolayer was stained with crystal violet and the plaque diameter measured using Axiovision 4.6 software and a Zeiss Axiovert 200 M microscope. Results are expressed as the mean plaque radius ± SD. (TIF) [file ppat.1003183.s002.tif]

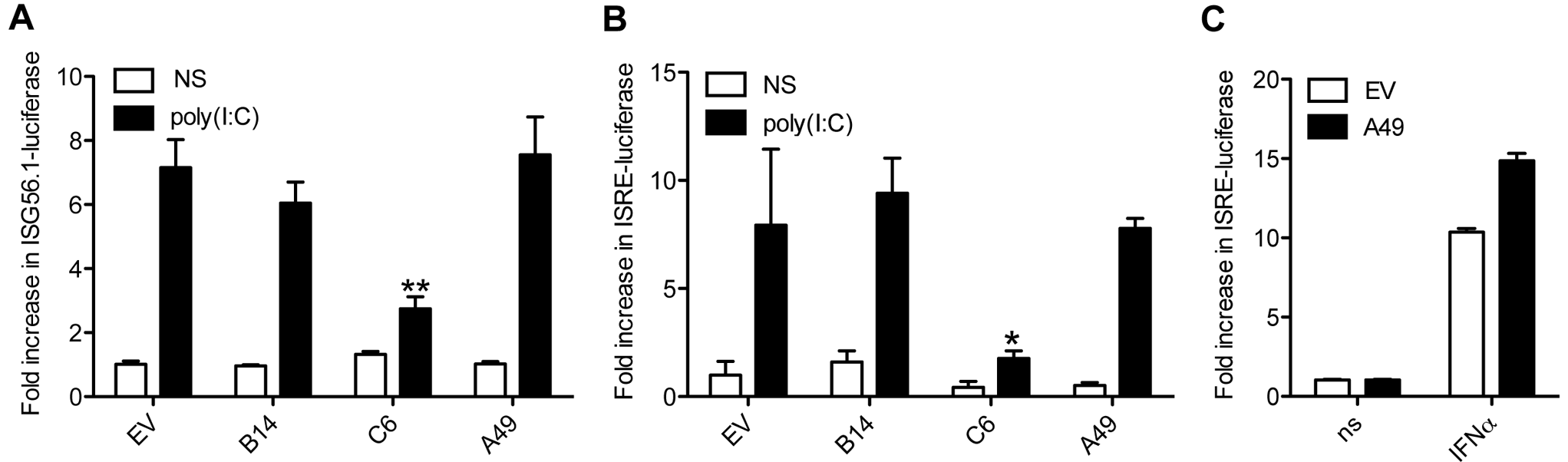

Supplement: Figure S3 — A49 does not inhibit ISRE or IRF3 activation. (A–B) HEK293ET cells were transfected with empty vector (EV), B14, C6 or A49, together with a renilla luciferase and (A) the IRF3-specific reporter ISG56.1-Luc or (B) an ISRE-Luc. After 24 h cells were stimulated with poly(I∶C) for 6 h and the luciferase activity was measured. (C) HeLa cells were transfected with pCI-A49 or empty plasmid (EV), an ISRE-Luc reporter and the TK-renilla control. After 24 h cells were treated with 500 u/ml of IFNα for 6 h and the luciferase activity was measured. Data are presented as mean ± SD and show one representative experiment of at least three, each performed in triplicate. *p<0.05 or ** p<0.01 comparing A49, C6 or B14 transfected cells with EV. (TIF) [file ppat.1003183.s003.tif]

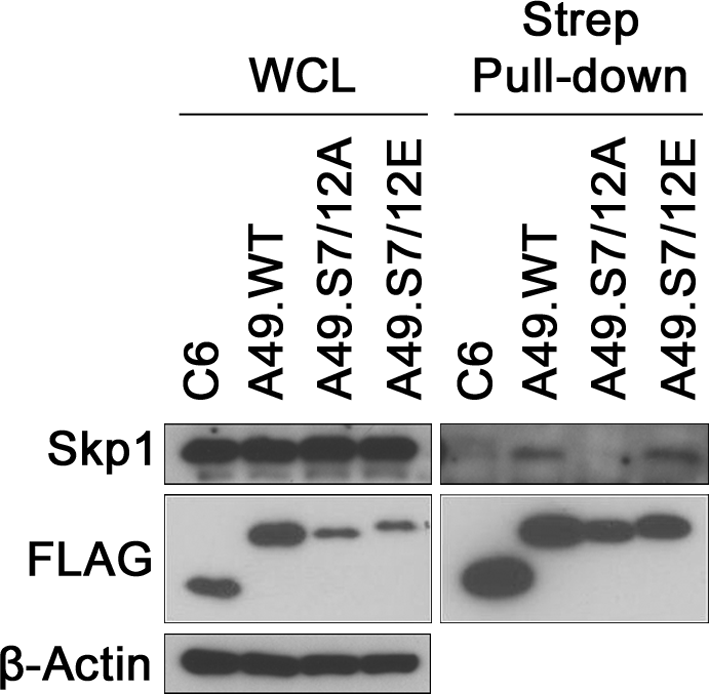

Supplement: Figure S4 — A49 binds the SCF machinery via β-TrCP. HeLa cells were transfected with plasmids encoding WT A49, or mutants S7/12A A49 or S7/12E A49, or VACV protein C6. Each VACV protein was fused at the N-terminus with a TAP tag. After 24 h, cells were lysed in IP buffer and a streptavidin pull-down was performed. Samples were analysed by SDS-PAGE and immunoblotting with the indicated antibodies. Whole cell lysate (WCL, 2%) of each sample was loaded. (TIF) [file ppat.1003183.s004.tif]

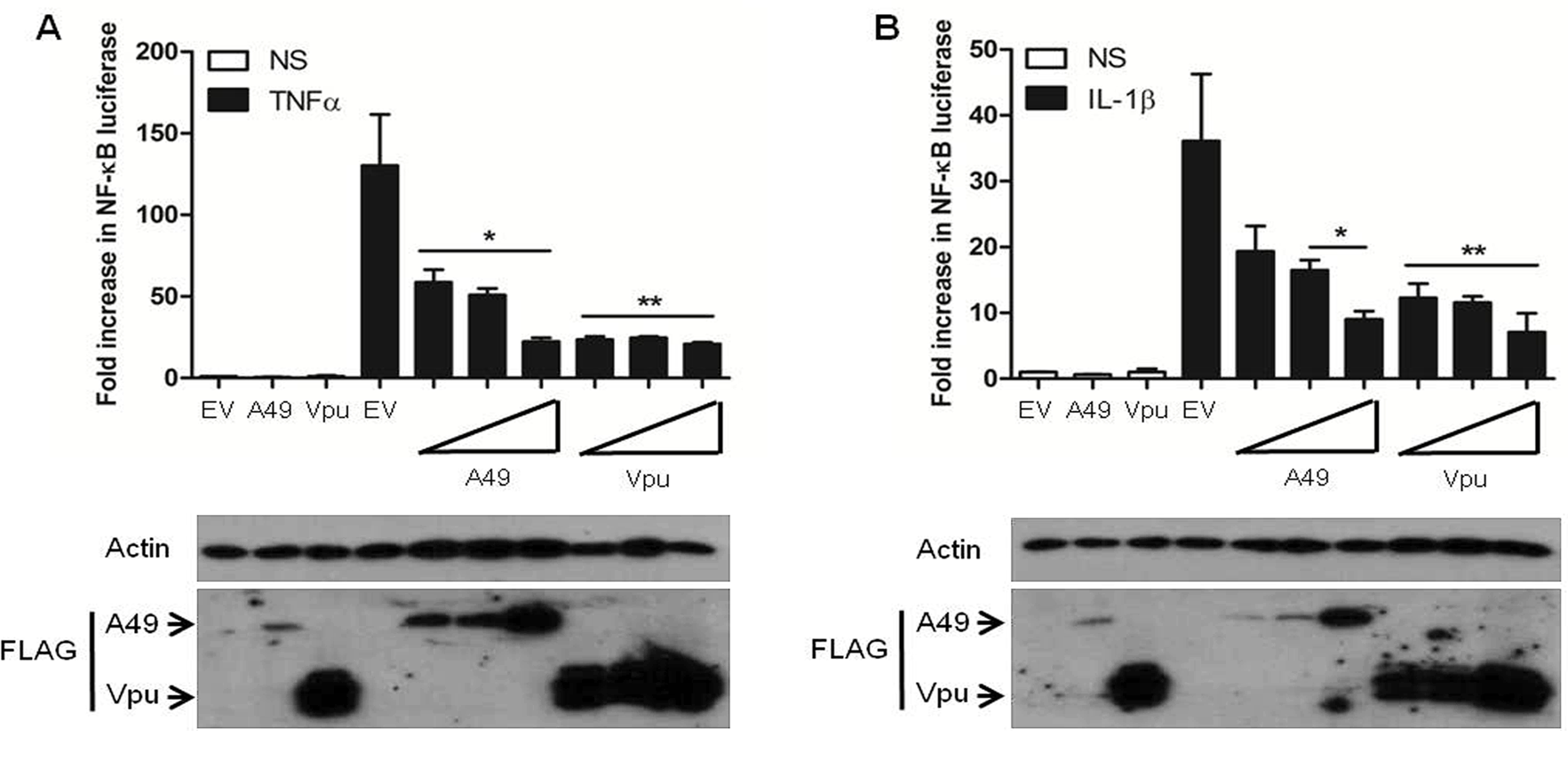

Supplement: Figure S5 — Inhibition of NF-κB activation by A49 and Vpu. (A–B) HEK293T cells were transfected with empty vector (EV), of plasmids expressing A49 or Vpu, together with a renilla luciferase and the NF-κB-Luc reporter. After 24 h cells were stimulated with TNFα (A) or IL-1β (B) for 6 h and the luciferase activity was measured. Whole cell lysate (12.5%) of each sample was analysed by SDS-PAGE and immunoblotted for FLAG and actin. Data are presented as mean ± SD and show one representative experiment of at least three, each performed in triplicate. *p<0.05 or ** p<0.01 comparing A49 or Vpu transfected cells with EV. (TIF) [file ppat.1003183.s005.tif]

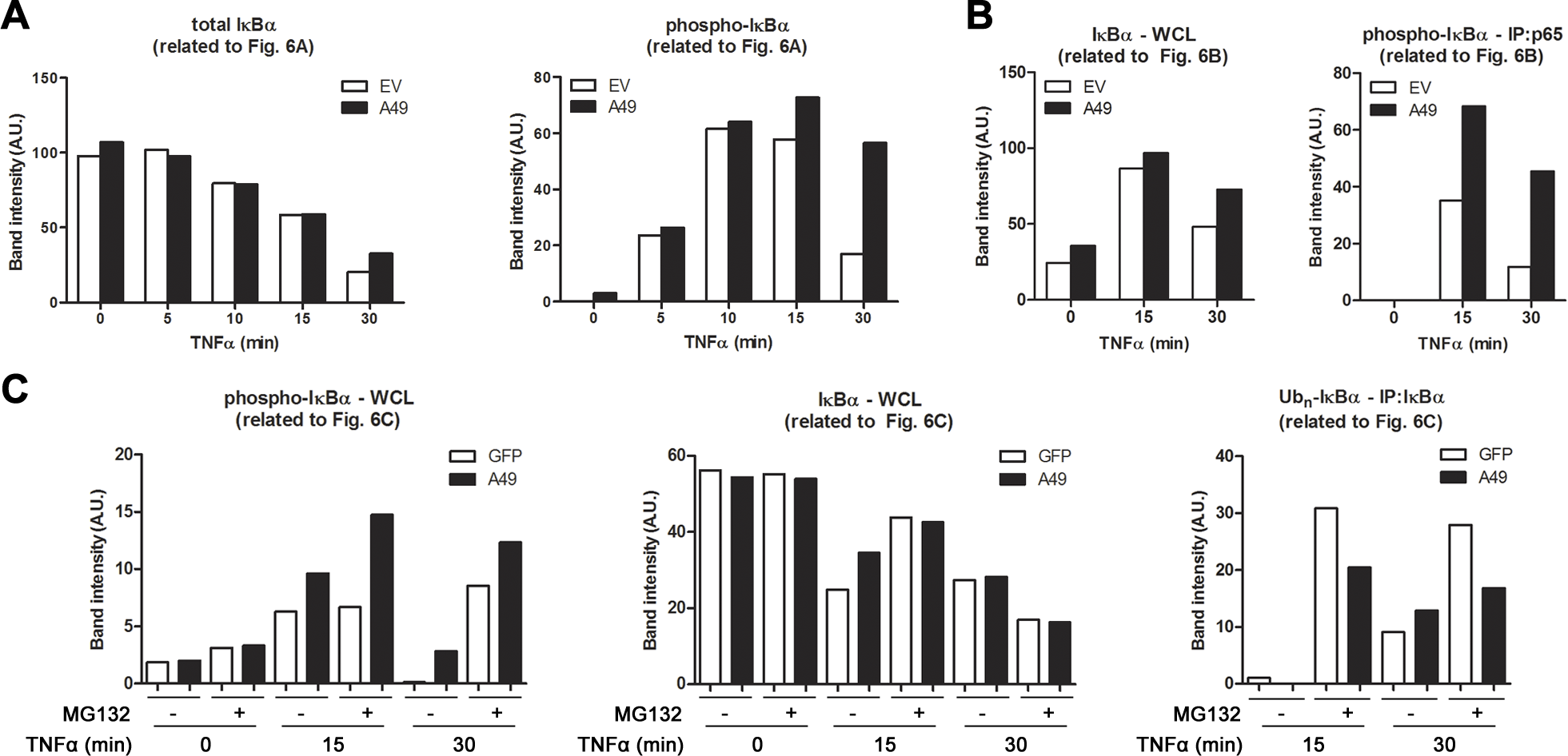

Supplement: Figure S6 — Quantitation of p-IκBα, IκBα, and ubiquitinated IκBα, by densitometry. Intensity of the bands corresponding to p-IκBα, IκBα (A–B) and ubiquitinated IκBα C) from pictures shown in Figure 6 was analysed by ImageJ and represented as bars in its arbitrary units (AU) after subtracting background signal. (TIF) [file ppat.1003183.s006.tif]

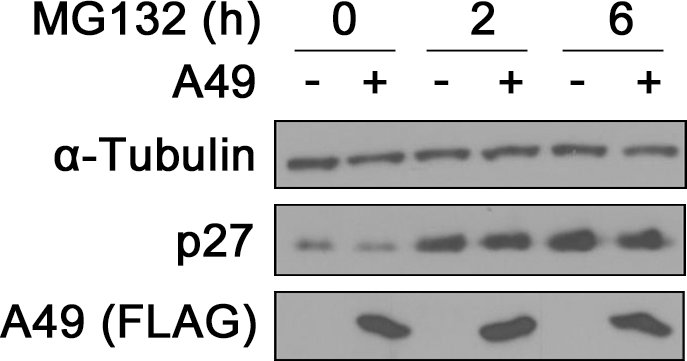

Supplement: Figure S7 — A49 does not affect the proteasome non-specifically. HeLa cells were transfected with pCI-A49 (A49) or the empty vector and, 24 h later, were treated with MG132 (20 µM) for different lengths of time. Cell extracts (4% of total) were prepared separated by SDS-PAGE and analysed by immunoblotting with the antibodies indicated. (TIF) [file ppat.1003183.s007.tif]
